# Pyrophosphate therapy prevents trauma‐induced calcification in the mouse model of neurogenic heterotopic ossification

**DOI:** 10.1111/jcmm.15793

**Published:** 2020-09-04

**Authors:** Natália Tőkési, Eszter Kozák, Krisztina Fülöp, Dóra Dedinszki, Nikolett Hegedűs, Bálint Király, Krisztián Szigeti, Kitti Ajtay, Zoltán Jakus, Jeremy Zaworski, Emmanuel Letavernier, Viola Pomozi, András Váradi

**Affiliations:** ^1^ Institute of Enzymology Research Center for Natural Sciences Hungarian Academy of Sciences Centre of Excellence Budapest Hungary; ^2^ Department of Biophysics and Radiation Biology Semmelweis University Budapest Hungary; ^3^ Department of Cellular and Network Neurobiology Institute of Experimental Medicine Budapest Hungary; ^4^ Department of Physiology Semmelweis University Budapest Hungary; ^5^ Sorbonne Université UPMC Univ Paris 06 Paris France; ^6^ INSERM UMR S 1155 Paris France

**Keywords:** animal models, bone‐brain‐nervous system interactions, disorders of calcium/phosphate metabolism, intervention, preclinical studies

## Abstract

Trauma‐induced calcification is the pathological consequence of complex injuries which often affect the central nervous system and other parts of the body simultaneously. We demonstrated by an animal model recapitulating the calcification of the above condition that adrenaline transmits the stress signal of brain injury to the calcifying tissues. We have also found that although the level of plasma pyrophosphate, the endogenous inhibitor of calcification, was normal in calcifying animals, it could not counteract the acute calcification. However, externally added pyrophosphate inhibited calcification even when it was administered after the complex injuries. Our finding suggests a potentially powerful clinical intervention of calcification triggered by polytrauma injuries which has no effective treatment.

## 1 INTRODUCTION

Several kinds of trauma can trigger heterotopic calcification in soft tissues (trauma‐induced calcification, TIC) involving the injury of the brain or the spinal cord, wounds, burn, bone fracture and orthopaedic operations or the combination of thereof. The calcification pathology in a later stage usually results in the formation of mature lamellar bone in the extraskeletal tissues (heterotopic ossification, HO). These pathological calcifications cause complex clinical problems with no other intervention than anti‐inflammatory medication and/or repeated surgical removal combined with radiation. The molecular and cellular events leading to HO are not understood in detail; for a recent review, see Zhang et al.^1^

Formation of TIC and HO is associated with a systemic and localized strong inflammatory response,^2^ the released cytokines trigger the differentiation of progenitor stem cells into osteoprogenitors and then the generation of new biomineral deposits and finally often new bone‐like structures.^3^ The osteo‐differentiation is manifested in the dysregulation of several signalling networks, the major “player” is the bone morphogenic proteins (BMPs)‐mediated pathway; reviewed by.^4^ These regulatory effects lead to the activation of transcription factors as Sox‐5, 6, 9, Runx2.^5^

In the present report, we focus on the typical case of a complex trauma‐induced calcification when the patient suffers from both injury of the central nervous system (CNS) and wound in the muscle (severe military, traffic or sport accidents; neurogenic heterotopic ossification).^6^ We have established an animal model with no calcification when either trauma‐induced brain injury or muscle damage was introduced separately, however, the simultaneous execution of the two resulted in massive calcification in the muscle. We investigated the role of the sympathetic nervous system in transmitting the stress of the brain injury to the calcifying tissues. Furthermore, we have tested the effect of the inhibitory metabolite, pyrophosphate on the complex trauma‐induced calcification.

## 2 MATERIALS AND METHODS

### 2.1 Study approval and animals

The animal studies have been approved by the Ethical Committee of Animal Experiments, Governmental Office of Pest County, Hungary; No. PE/EA/280‐7/2019 and were conducted according to the national guidelines. All animals were housed in approved animal facilities at the Research Centre for Natural Sciences, Hungarian Academy of Sciences. Mice were kept under routine laboratory conditions with 12‐hour light‐dark cycle with ad libitum access to water and chow. CD1 wild‐type female mice were obtained from Ttw^+/−^ (kind gift of Prof. Frank Rutsch, University of Münster, Germany) mating and Enpp1^+/+^ genotype was confirmed in each animal. C57BL/6J mice were derived from the Department of Experimental Pharmacology, National Institute of Oncology, Hungary. Balb/C mice were obtained from the Immunology Department of Eötvös Loránd Science University, Hungary.

### 2.2 Anaesthesia

Anaesthesia was carried out by intraperitoneal injection of the mixture of Zoletil (30 mg/kg, Virbac, France), Xilazin (12.5 mg/kg, Produlab Pharma, The Netherland) and Butorfanol (3 mg/kg, Richterpharma, Austria).

### 2.3 Traumatic brain injury (TBI)

Mice (24‐32 g, female) were subjected to TBI in anaesthesia. A weight‐drop device was used to induce TBI as described.^7^ A weight (19 mm diameter, 95 g) of solid brass was utilized, a small steel cap (2 × 10 mm) was glued to the bottom of the weight to restrict the zone of contact to the top of the mouse head. Weight is dropped vertically through a guiding tube (20 mm diameter × 1.5 m length). The vertical traverse of the dropped weight is limited to ∼40 mm of impact displacement. A stage consisting of aluminium foil holds the animal in place. In this fashion, the foil supports the bodyweight with little resistance but upon impact (TBI) it breaks. The animals land on a sponge cushion.

### 2.4 Cardiotoxin (CTX) injury

To induce muscle injury, immediately after TBI still in anaesthesia, mice (24‐32 g, female) received a single injection of CTX into the hamstring muscle (0.12‐0.16 mg/kg in 50 μL from a 12 μM solution of CTX; L8102, Latoxan, France; dissolved in sterile saline), which was delivered using a lateral approach using a 26G needle attached to an insulin syringe.

### 2.5 Plasma adrenaline measurement

Plasma adrenaline level was measured by the Epinephrine/Norepinephrine ELISA kit (KA1877, Abnova) according to the manufacturer's instructions. Blood was taken by cardiac puncture.

### 2.6 Adrenaline and adrenaline‐receptor antagonist

Adrenaline (Tonogen, Richter Gedeon, Hungary), 2 mg/kg, was injected intraperitoneally 5 minutes before CTX injection. Adrenaline‐receptor antagonist prazosin (5 mg/kg, P7791, Sigma), yohimbine (1 mg/kg, Y3125, Sigma) and propranolol (3 mg/kg, P0884, Sigma) were dissolved in sterile saline and injected intraperitoneally 15 minutes before TBI.

### 2.7 Histochemistry

Calcification of the muscle was visualized by Alizarin Red and von Kossa histochemistry following described methods.^8^

### 2.8 CT scanning and analysis

CT measurements were performed on a NanoX‐CT (Mediso, Hungary) cone‐beam micro‐CT imaging system. Circular CT scans were acquired of two samples at a time with an 8W power X‐ray source with 55 kV tube voltage, 1.36 magnification, 900 ms exposure time, 1:1 binning and 360 projections in 7 minutes. For reconstruction, we used filtered back projection with a Butterworth filter, and the isotropic voxel size was set to 70 μm. CT images were loaded into the open‐source 3D Slicer software^9^ (http://www.slicer.org), and a semi‐automatic segmentation procedure was carried out to partition calcified tissue which has a signal intensity within the range induced by bone structure (maximum) and soft tissue (minimum). First, areas containing calcified tissue were localized manually. Second, a local intensity threshold was applied to these areas to select the actual region of interest, but not the similar intensity noise from other regions of the image. As a final step, the volume of the segmented areas was measured, and a 3D model was created for visualization purposes with the built in tools of the software. For visualizing bone structures in the 3D figures, we used the volume rendering module of the software.

### 2.9 Microcalcifications

Microcalcifications were characterized using Fourier transform infrared microspectroscopy (µ‐FTIR). Tissue sections (4‐µm) were deposited on low‐emission microscope slides (MirrIR, Keveley Technologies, Tienta Sciences, Indianapolis). FTIR hyperspectral images were recorded with a Spectrum spotlight 400 FTIR imaging system (Perkin Elmer Life Sciences, Courtaboeuf, France), with a spatial resolution of 6.25 micrometre and a spectral resolution of 8 cm^‐1^. Each spectral image covering a substantial part of the tissue consisted of about 30,000 spectra.

### 2.10 Gene expression analysis

Total RNA was extracted from ∼30 mg muscle and liver tissues using TRIzol reagent (Thermo Fisher Scientific, MA, USA), and cDNA was synthesized using the SuperScript III First‐Strand synthesis kit with random hexamers (Thermo Fisher Scientific, MA, USA). In each reaction, 1 μg total RNA was used. The synthesis was performed as recommended by the manufacturer.

RT‐qPCR was carried out using the Applied Biosystems StepOnePlus RT‐PCR system and the Design Wizard StepOne software (Applied Biosciences Inc, CA, USA). Expression level of Abcc6 (Mm00497698_m1) and Enpp1 (Mm01193761_m1) in liver and Runx2 (Mm00501584_m1), Sox9 (Mm00448840_m1), Bmp‐2 (Mm01340178_m1) and Bmp‐4 (Mm00432087_m1) in muscle was detected using commercially available TaqMan probes (Thermo Fisher Scientific, MA, USA). The expression level of Abcc6 and Enpp1 in liver was normalized to beta2microglobulin (Mm00437762_m1). In muscle tissue, the expression level of four housekeeping genes: Gapdh (Mm99999915_g1), Actin‐b (Mm00607939_s1), Hmbs (Mm01143545_m1) and ®2m(Mm00437762_m1) was found unstable between the different treatment groups, and therefore, they were not used as reference genes, rather we have normalized the obtained expression data to total amount of RNA.

### 2.11 Pyrophosphate treatment and measurement

Mice received combined PPi treatment. On one hand, mice were treated with 80‐100 mg/kg Na_4_P_2_O_7_ (71515, Sigma) dissolved in sterile saline via single daily intraperitoneal (IP) injections. The first IP was added right after TBI + CTX or ADR + CTX treatment, except in TBI + CTX+30 min PPi group, where it was added 30 min after TBI + CTX. Besides these IP injections, mice were simultaneously treated orally with PPi by changing their drinking water to 1 mM Na_4_P_2_O_7_ dissolved in distilled water after TBI. This combined PPi treatment was continued for 4 days. Control group received distilled water during the experiment. Determination of PPi concentration in plasma was performed as described in our previous paper.^10^

### 2.12 Statistical analysis

Data were analysed by two‐tailed Mann‐Whitney non‐parametric test. Values are expressed as mean and standard error of the mean (SEM). A *P* < .05 was considered statistically significant, and the actual p‐values are indicated on the corresponding figures. Animal numbers used for individual data sets varied and are shown in the figures.

## 3 RESULTS

### 3.1 Combined traumatic brain injury and skeletal muscle damage (TBI + CTX), a model of neurological trauma‐induced calcification

**FIGURE 1:**
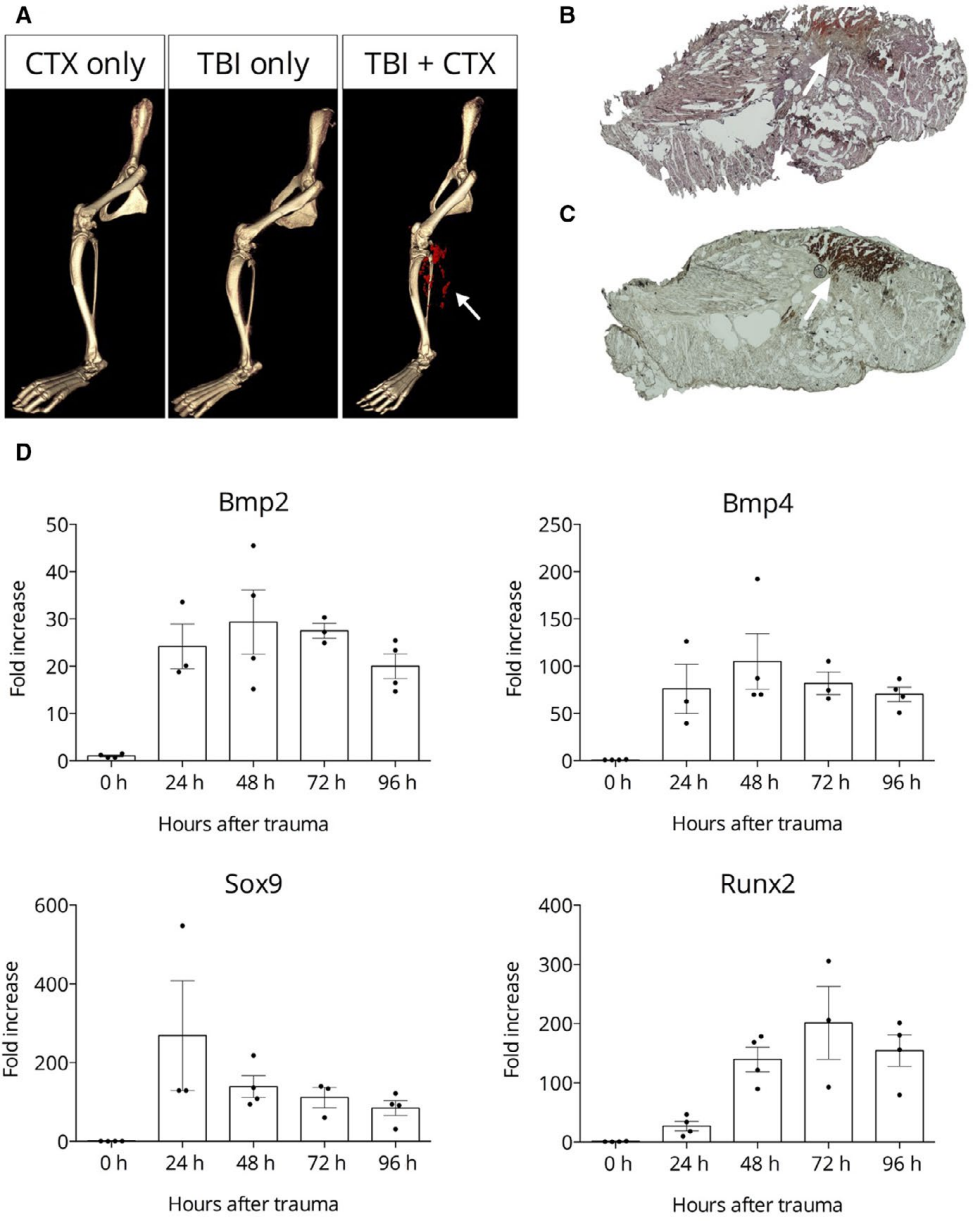
Calcification of the hamstring muscle upon complex injury. Panel A: Representative micro‐CT images, white arrows point to the calcified area (red). Panels B and C: Representative histochemistry of the calcified muscle from a TBI + CTX‐treated animal: Alizarin Red staining for calcium (B) and von Kossa staining for phosphate (C). The white arrows point to the calcified tissue. Panel D: Expression kinetics of genes *Bmp2, Bmp4, Runx2* and *Sox9* upon trauma‐induced calcification in hamstring muscle

Spinal cord injury combined with skeletal (hamstring) muscle damage introduced by cardiotoxin injection has been described as an animal model of TIC.^11^, ^12^ We have modified the original Genet‐Torossian method and instead of trans‐section of the spinal cord we have introduced a mild mechanical (traumatic) injury of the brain (TBI),^7^ and combined this intervention with cardiotoxin‐induced damage (CTX) in the hamstring muscle. We have observed no calcification when either TBI or CTX was introduced alone, however the simultaneous execution of the two insults resulted in massive calcification in the hamstring muscle, similar to the previously described, but more radical intervention. We have tested our novel approach (TBI + CTX) on three different mouse strains, C57Bl6, Balb/C and CD1. All three reacted unequivocally on the same manner: robust calcification in the injured hamstring muscle after four days of the combined intervention. The calcification of CD1 animals (we used these animals in our further studies) was detected by micro‐CT (Figure 1 panel A). Histochemistry of the hamstring muscle indicates strong Alizarin Red and von Kossa‐positive calcified deposits (Figure 1, panels B and C, respectively).

### 3.2 Expression of “calcification” genes in calcifying muscle

Next, we asked whether the trauma‐induced calcification we are studying follows the usual route of the known osteo‐differentiation pathway. In order to answer this question, we have collected hamstring muscle samples from animals on days 1, 2, 3 and 4 after TBI + CTX and compared the values to that of without TBI + CTX. We determined the expression level of genes *Bmp2* and *Bmp4*, as well as *Runx2* and *Sox 9*. In this experiment, we faced a technical difficulty: as we were studying heavily damaged tissue with dying and with de novo cells we could not use a reference gene (“housekeeping gene”), since the expression of *Gapdh* or *Actin*, or *beta2microglobulin*, or *Hmbs* genes was not stable during the four days calcification period. Therefore, we have normalized the obtained expression data to total amount of RNA. The expression kinetics from all four genes showed a similar pattern: their abundance was barely detectable in control, non‐injured animals and increased dramatically at day 1 through day 4 (Figure 1D).

### 3.3 Adrenaline mediates calcification

**FIGURE 2:**
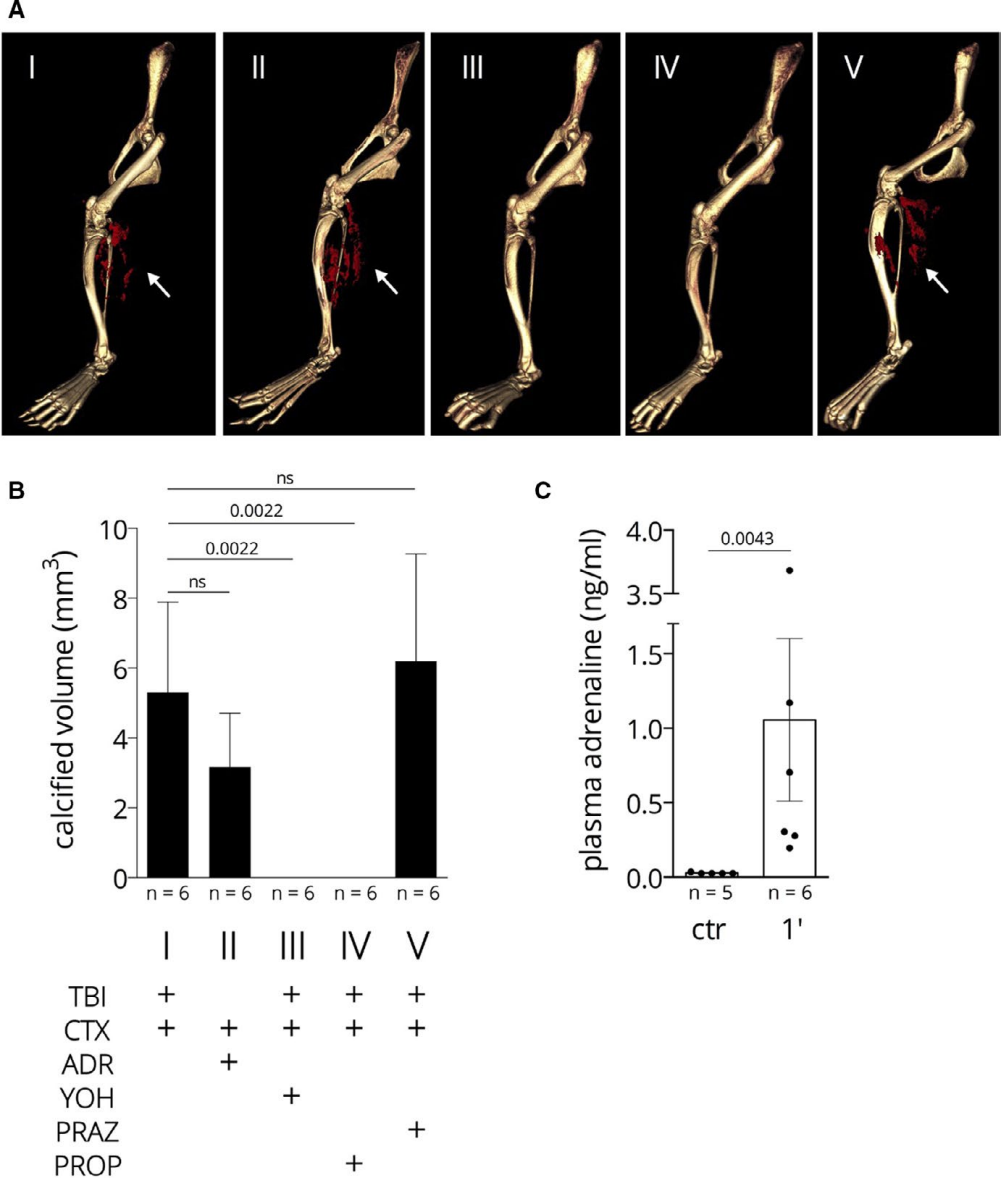
Calcification of the hamstring muscle upon complex injury; effect of adrenaline and adrenaline‐receptor antagonists. Panel A: Representative micro‐CT images, white arrows point to the calcified area (red). Panel B: Quantitative determination of the mineral deposits by micro‐CT volume measurement. Panel C: plasma adrenaline concentration before (ctr) and 1 min after TBI

As muscle injury (CTX) without traumatic brain injury (TBI) did not trigger calcification in the injured muscle, we hypothesized that the reaction to the CNS‐directed stress is transmitted through the sympathetic nervous system. Therefore, we wanted to test whether increased plasma level of adrenaline due to the CNS shock does play a role in “transmitting” the TBI stress to the injured muscle. Indeed, we could measure a large increase of plasma adrenaline level 1 min. after TBI (see Figure 2, panel C). Then, we replaced TBI with injecting adrenaline intraperitoneally (ADR + CTX approach), 2 mg/kg, 5′ before CTX. As a control, we have applied adrenaline injection alone which resulted in no calcification in the hamstring muscle (similar to the lack of effect of TBI alone, see above). However, ADR + CTX resulted in massive calcification to the same extent as TBI + CTX: 3.04 mm^3^ vs 5.31 mm^3^, the two data sets are not significantly different (see Figure 2. Panel A Images I and II and panel B). These results support that adrenaline plays a crucial role in the stress‐mediated communication between CNS and the calcifying skeletal muscle.

### 3.4 Effect of adrenaline‐receptor antagonists

Next, we addressed the question: what is the target receptor of the stress signal on the calcification pathway, and therefore, we treated mice with adrenaline‐receptor antagonists 15 min before TBI + CTX. We have injected yohimbine, an alpha2 receptor antagonist intraperitoneally (1 mg/kg) and observed a massive inhibition of calcification compared to the experiment without yohimbine (0 vs 5.31 mm calcified deposit in the hamstrings, *P* = .009, see Figure 2 panel A, images I and III and panel B). These results point to alpha2 adrenergic receptor playing role in TBI‐induced calcification. Prazosin (5 mg/kg), an alpha1‐receptor antagonist was not effective in halting TBI + CTX‐induced calcification (5.31 vs 8.96, the two data set are not significantly different, see Figure 2 panel A, images I and V and panel B). The beta‐receptor antagonist propranolol (3 mg/kg) inhibited calcification (5.31 vs 0) on the same way as yohimbine (5.31 vs 0 mm^3^ calcified deposit; see Figure 2 panel A, images I and IV and panel B.) The results of the experiments with adrenaline‐receptor antagonists argue that alpha2 and beta‐adrenergic receptors play a role in TBI‐induced calcification, while alpha1 receptor probably does not participate in transmitting the stress signal.

### 3.5 Neither plasma pyrophosphate level nor the expression of hepatic proteins controlling plasma PPi change upon TBI + CTX

**FIGURE 3:**
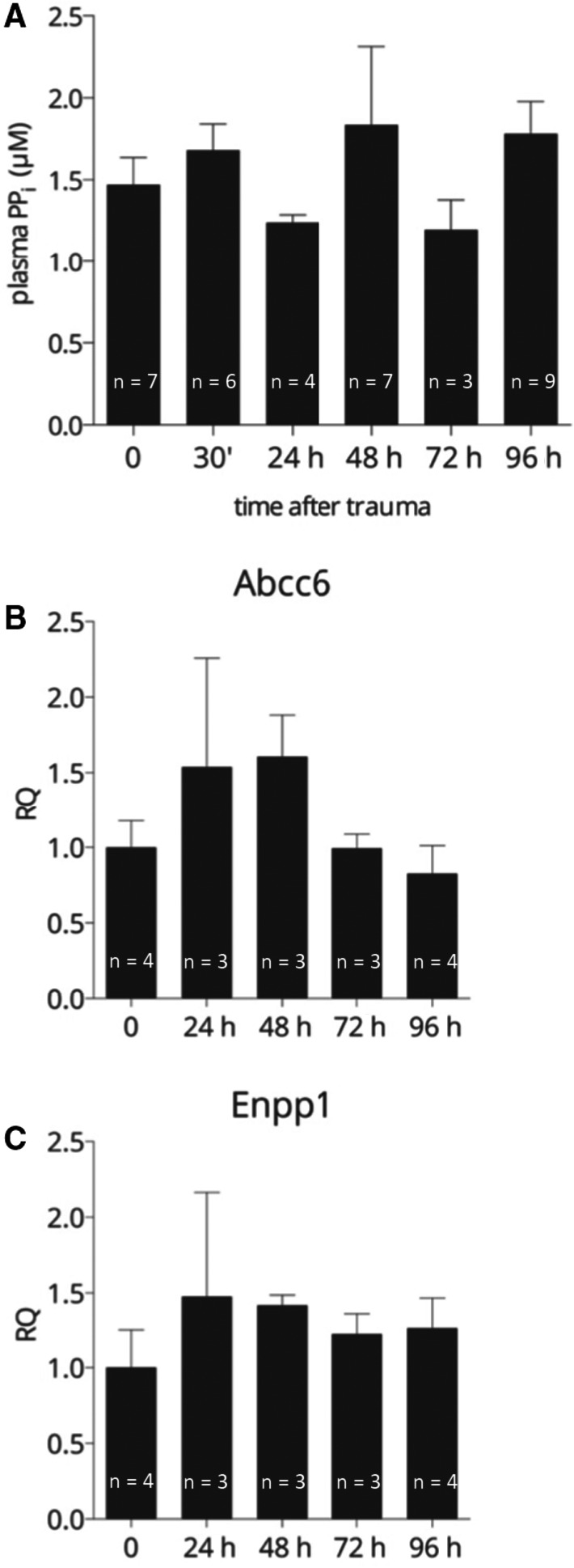
Plasma pyrophosphate level and expression of genes *Abcc6* and *Enpp1* in the liver during trauma‐induced calcification. A: Plasma pyrophosphate level before (0) and at different time‐points after TBI + CTX. B and C: Expression of genes in the liver controlling plasma pyrophosphate level (B: *Abcc6*, C: *Enpp1*). Expression levels were normalized to that of beta2microglobulin

As reduced plasma pyrophosphate level is the metabolic cause of inherited ectopic calcification disorders pseudoxanthoma elasticum (PXE) and generalized arterial calcification in infancy (GACI), we have supposed that reduction of PPi in plasma is the cause of TBI + CTX‐induced calcification too. To test this hypothesis, we have measured plasma pyrophosphate concentration in TBI + CTX (ie in calcifying animals) at 1, 2, 3 and 4 days and at 30′ after the combined trauma and compared the values to that of without TBI + CTX. We have observed that plasma PPi levels did not change significantly during calcification in the hamstring muscle (Figure 3, panel A).

The main source of circulating pyrophosphate is the liver and mutations in any of the two key proteins responsible for plasma PPi level, in ABCC6 or ENPP1 result in low level of plasma PPi. We have measured the expression of *Enpp1* and *Abcc6* genes in the liver of animals upon TBI + CTX at days 1, 2, 3 and 4 and compared the values to those of without TBI + CTX. We could not detect altered expression of either *Abcc6* or *Enpp1* genes in the liver (Figure 3, panels B and C). This finding is in harmony with the unaffected plasma pyrophosphate levels of these animals.

### 3.6 Externally added pyrophosphate inhibits trauma‐induced calcification

**FIGURE 4:**
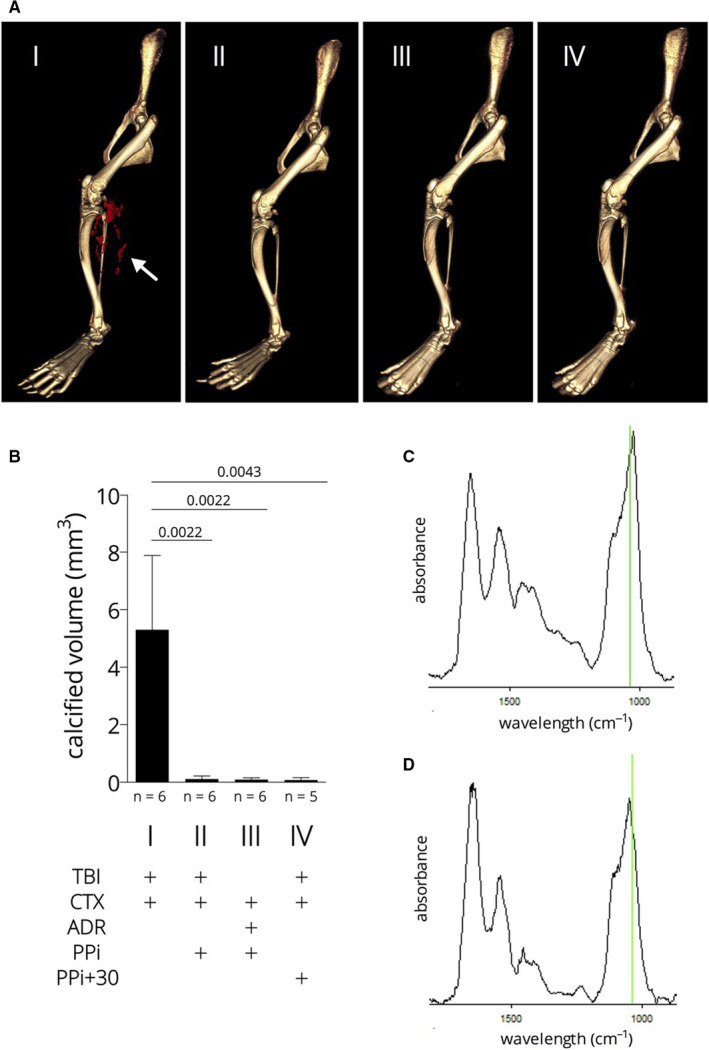
Calcification of the hamstring muscle upon complex injury; effect of pyrophosphate. Panel A: micro‐CT images, white arrow points to the mineral deposits (red). Panel B: quantitative determination of the mineral deposits by micro‐CT volume measurement. Panel C and D: representative Fourier transform infrared microspectroscopy (μ‐FTIR) spectra of microcalcifications of the hamstring muscle of a TBI + CTX animal (C) and a TBI + CTX animal treated with pyrophosphate (D). Green lines correspond to 1030 cm^−1^, the ν3 P–O stretching vibration mode of apatite

We found that normal level of plasma pyrophosphate is not sufficient to counteract the rapid calcification due to the complex trauma, and therefore, we tested the effect of externally added pyrophosphate. Mice subjected to the TBI + CTX were put on combined PPi treatment: Addition of 1 mM Na_4_P_2_O_7_ in drinking water was combined with single daily intraperitoneal injections of Na_4_P_2_O_7_, 80‐100 mg/kg for four days. The first IP injection was added right after TBI (within 1 min). This treatment resulted in a very effective inhibition of mineral deposition in the hamstring muscle (Figure 4 Panel A image I and II and Panel B): 5.25 mm^3^ vs 0.11 mm^3^ with a p value of 0.02 (PPi in drinking water alone was not effective, not shown). This PPi treatment was found to be highly effective in inhibiting ADR + CTX‐induced calcification too; 3.04 mm^3^ vs 0.09 mm^3^, *P* = .03 (see Figure 4 Panel A image I. and III. and Panel B). This is similar to the effect of PPi on the TBI + CTX calcification (the two data sets are not different, *P* = .40). This finding supports that the step(s) of hydroxyapatite crystal formation inhibited by PPi is/are similar in the TBI and ADR‐induced pathways.

As PPi is capable of inhibiting calcification, we raised the question: can PPi be effective on the TBI + CTX‐induced calcification if the treatment is not started immediately after the injury? Therefore, we have applied the first intraperitoneal PPi injection 30 min after the TBI + CTX injury, thus mimicking emergency intervention after a severe traffic or combat injury. Otherwise, the PPi treatment was the same as above and continued for four days. We have observed a very effective inhibition of calcification, 5.25 mm^3^ vs 0.08 mm^3^ with a p value of 0.03 (see Figure 4 Panel A image I and IV and Panel B). The microstructure of the mineral deposit in the pyrophosphate‐treated animals was not different from that developed in non‐treated animals (see Figure 4, panels C and D). It showed the typical composition of ectopic mineralization deposits: carbonated apatite with no octacalcium phosphate (OCP) and with low amorphous calcium‐phosphate levels. It is obvious that PPi rather changes the volume and not the structure/composition of the mineral deposits.

## 4 DISCUSSION

Pathological ectopic calcification of soft (connective) tissues is a major clinical problem, what is often associated with multi‐traumatic injuries. The trauma involves a wide variation of impacts including injuries of the CNS, large burns, fracture of bones and major surgical interventions. It was recently demonstrated that deposition of hydroxyapatite within damaged skeletal muscle is sufficient to predispose skeletal muscle to HO indicating that dystrophic and heterotopic calcifications may be stages of a pathologic continuum.^13^ Approximately, 90% of TBI patients with a fracture or dislocation of the elbow develop heterotopic ossification.^14^ The prevalence of calcification leading to severe heterotopic ossification is 64.5% in combat‐related polytrauma injuries.^15^ Our in vivo model is based on a mild mechanical injury of the brain (TBI)^7^ combined with one of the most reliable muscle injury models resulting in pathophysiological events that occur in human muscle, with the cardiotoxin‐induced damage of hamstring muscle (CTX). We have observed calcification in the hamstring muscle only when TBI and CTX were introduced simultaneously. This observation supports that our animal model recapitulates the details of severe military‐, traffic‐ or sport accident‐triggered neurological heterotopic ossification.^6^

We have asked the question: how stress is transmitted from the CNS to the calcifying tissue and found that adrenaline with concomitant CTX induces calcification in the injured muscle as effectively as the brain trauma indicating the role of the sympathetic nervous system in signalling. We have also found that adrenergic receptors alpha2 and beta are involved in the stress‐mediated communication as pharmacological block of those receptors suspended the adrenaline‐effect while alpha1 receptors seem not to be involved. The observation that blocking adrenergic receptors inhibits calcification suggested a therapeutic intervention using antagonists. However, we found no calcification inhibition if the alpha2 blocker yohimbine was added 30 minutes after the complex injury (10 mg/kg; not shown), this finding does not support an intervention based on adrenergic receptor blockage.

Up‐regulation of transcription from the genes of the key protein components of tissue calcification, *Bmp2, Bmp4, Runx2, Sox9* was observed using qRT‐PCR assay. All the above features indicate that the TBI + CTX polytrauma method triggers calcium‐phosphate/hydroxyapatite precipitation on the well‐known inflammatory‐osteo‐differentiation pathway.

Our major finding was that externally added pyrophosphate, an endogenous inhibitor of hydroxyapatite formation ^16^ inhibits the complex trauma‐induced calcification, even when it is added post‐trauma. It is worth to note that the microstructure of the mineral deposit in the pyrophosphate‐treated animals was not different from that developed in non‐treated animals (see Figure 4, panels C and D), that is PPi rather changes the volume and not the structure/composition of the mineral deposits. PPi inhibits the ADR + CTX‐induced calcification in the same way, further supporting that the adrenaline‐induced calcification is similar to that of the polytrauma‐induced calcification.

Our next intention was to uncover the background of PPi action. We could not detect significant change in the plasma PPi level during the 4‐day period of calcification when the values were compared to those prior polytrauma. The main source of plasma pyrophosphate is the liver, and the initial step is the ABCC6‐facilitated release of trinucleotides, mostly ATP from hepatocytes. ATP is converted promptly in the liver microcirculation to AMP and PPi by the ectonuclease, ENPP1.^16^, ^17^ Mutations in any of the two key proteins result in decreased plasma PPi level, what is the metabolic cause of two monogenic inherited calcification diseases, pseudoxanthoma elasticum and generalized arterial calcification in infancy; for a review, see Borst et al.^18^ We have determined the transcription from genes *Abcc6* and *Enpp1* in the liver by qRT‐PCR before polytrauma and during the 4 days calcification and found no significant changes in the abundance of either mRNA (compared to the values obtained from livers before polytrauma treatment). These findings are in harmony with the observation of no significant change in the plasma PPi level during calcification. We can conclude that the key genes’ activities determining systemic PPi and the plasma PPi level itself are “normal”, that is not declined, and therefore, this can be ruled out as the cause of the polytrauma‐induced calcification.

It has been shown long ago that the mechanism of PPi inhibitory action is based on blocking new calcium‐phosphate crystal formation (ie de novo crystal formation) and also the growth of existing crystals by binding to the surface of hydroxyapatite,^19^, ^20^ what is considered as the final common pathway in the pathophysiology of soft tissue calcification. This is the “physico‐chemical” effect of other polyphosphates too.^21^ It was also postulated that “it is apparently impossible for any calcification to occur in a system which contains a constant, physiological concentration of inorganic pyrophosphate”.^22^ In contrast, our results argue that the physiological level of PPi in plasma upon trauma‐induced calcification does not suffice to prevent the massive and rapid hydroxyapatite formation in the damaged muscle in the acute situation. The benefit of increasing pyrophosphate level (directly or indirectly by modifying enzyme activities of generating or removing PPi) has been demonstrated in several animal models representing human diseases as PXE,^10^, ^23^, ^24^, ^25^ as GACI,^26^ or Hutchinson‐Gilford progeria syndrome.^27^ In each of the above cases, the low PPi level is the pathological cause of the calcification symptoms. However, trauma‐induced calcification is different as the externally added PPi does not substitute for the low level of this metabolite, it rather provides a highly elevated systemic PPi level what is able to counteract the rapid calcification triggered by the complex injury.

Trauma‐induced calcification has no effective treatment. Our finding that pyrophosphate can prevent mineralization due to complex trauma—even when it was administered 30 minutes after trauma—raises the possibility of its therapeutical utilization. This encouraging result points to the potential of an “emergency” pyrophosphate therapy to be executed shortly after the accident. Furthermore, pyrophosphate can be used as a preventive therapy adding prior to planned, large surgical interventions with the danger of heterotopic calcification and the treatment can be continued post‐operation during recovery. The risk of pyrophosphate treatment is probably negligible as it is widely used as a food additive and the Code for Federal Regulation by the FDA states: “*This substance is generally recognized as safe”*.

## CONFLICT OF INTEREST

Dóra Dedinszki and András Váradi filed a patent “Oral pyrophosphate for use in reducing tissue calcification” (WO2018052290).

## AUTHOR CONTRIBUTIONS

**Natália Tőkési:** Data curation (equal); Investigation (lead); Validation (lead); Visualization (lead). **Eszter Kozák:** Data curation (equal); Investigation (equal); Validation (equal); Visualization (equal). **Krisztina Fülöp:** Data curation (equal); Investigation (equal); Validation (equal). **Dóra Dedinszki:** Investigation (equal); Visualization (equal). **Nikolett Hegedűs:** Investigation (equal); Visualization (equal). **Bálint Király:** Data curation (equal); Investigation (equal); Visualization (equal). **Krisztián Szigeti:** Investigation (equal); Methodology (equal). **Kitti Ajtay:** Investigation (equal); Validation (equal). **Zoltán Jakus:** Data curation (equal); Investigation (equal); Supervision (supporting); Validation (equal). **Jeremy Zaworski:** Investigation (equal); Methodology (equal); Visualization (equal). **Emmanuel Letavernier:** Investigation (equal); Methodology (equal); Supervision (supporting). **Viola Pomozi:** Data curation (equal); Investigation (equal); Methodology (equal); Project administration (equal); Supervision (supporting). **Andras Varadi:** Conceptualization (lead); Data curation (equal); Supervision (lead); Writing‐original draft (lead); Writing‐review & editing (lead).

## Data Availability

The data that support the findings of this study are available from the corresponding author upon reasonable request.
